# Perioperative fracture-dislocation of the humeral head during a resurfacing hemiarthroplasty

**DOI:** 10.4103/0973-6042.41036

**Published:** 2008

**Authors:** L. Peidro, R. Plaza, S. Sastre

**Affiliations:** Hospital Clínic Barcelona, Orthopaedics Department, C/ Villarroel, 170, Esc 12.0, Barcelona 08036 Spain

**Keywords:** Perioperative fracture, reaming, resurfacing humeral head, shoulder prosthesis

## Abstract

We report a case of perioperative fracture-dislocation of the humeral head produced during the reaming for a resurfacing replacement hemiarthroplasty (RRH) in a 79-year old woman. This is a surgical complication not previously described in the literature for this type of prosthesis design. Resurfacing humeral head implant has been noted as a useful treatment for glenohumeral arthropathies, also in elderly people, with a very low incidence of complications. However, as we report, they are possible.

It is advisable that conventional stemmed implants could be available when RRH is performed.

## INTRODUCTION

Indications for a humeral resurfacing replacement hemiarthroplasty (RRH) are similar to those for stemmed hemiarthroplasty, including osteoarthritis with concentric erosion and ruptured rotator cuff in elderly people. The aim of this article is to describe a perioperative fracture of the humeral head, which has not been reported before.

We believe that this complication should be considered when RRH is used in elderly people.

## CASE REPORT

**Figure 1:**
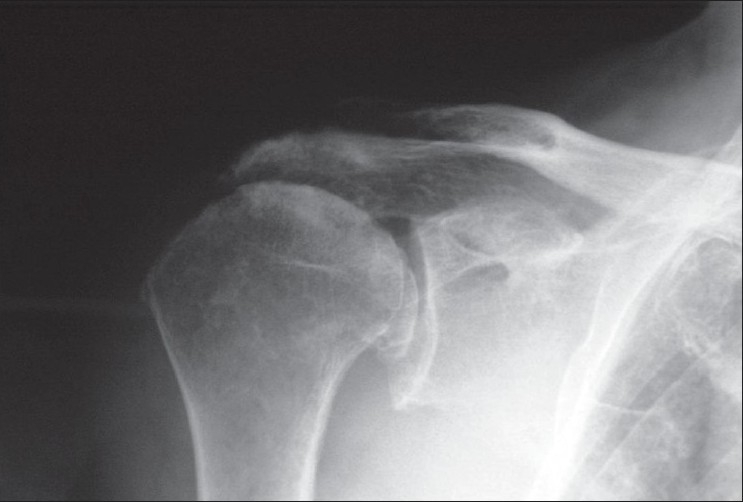
Preoperative X-ray showing osteoarthritis of the shoulder

**Figure 2:**
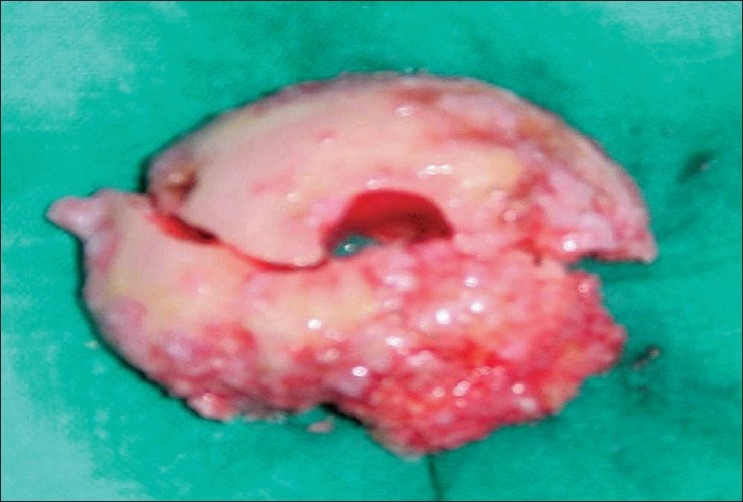
Fractured humeral head fragment

A 79- year- old woman with a history of severe cardiac insufficiency presented at our outpatient clinic with continuous bilateral shoulder pain and functional impairement of left predominance. Plain radiographs showed extensive osteoarthritis of both shoulders with reduced subacromial space [Figure 1]. We proposed a Reversed Shoulder Prosthesis but due to her very high surgical risk, finally, we decided to change to a Resurfacing Hemiarthroplasty. This procedure was initially performed with a Resurfacing Humeral Head implant (Global C.A.P..™., De Puy Orthopaedics), which had been recently introduced in our hospital. The reamer was new and the process was done in reaming speed as usual, after all the osteophytes had been excised. During this process, a sudden two-part anatomical neck fracture-dislocation of the humeral head occurred [Figure 2]. This complication has never been described for this surgical technique. Obviously, the resurfacing procedure was ruled out and a conventional modular stemmed hemiarthroplasty was placed uneventfully without worsening of the patient medical conditions.

## DISSCUSION

According to Copeland, indications for a (RRH) are similar to those for stemmed hemiarthroplasty, including osteoarthritis with concentric erosion and ruptured rotator cuff in elderly people as in our case.[1] Mean age of the patients treated with a Copeland Resurfacing Prosthesis was 68 years and it also included the diagnosis of rheumatoid arthritis.[2–4] Clinical experience with the Copeland cementless surface resurfacing arthroplasty (CSRA, Biomet) overcomes 20 years and a perioperative humeral head fracture-dislocation has not been reported with this implant.

The surgical experience with other resurfacing implants is limited but this complication has not been published either.[5,6] However, indications concerning the Global C.A.P. design are specially directed to osteoarthritis in young people, its results have not been published and its overall experience is short. In fact, it was our sixth case with this prosthesis, although the main surgeon experience on shoulder arthroplasties is quite long (since 1991).

**Figure 3:**
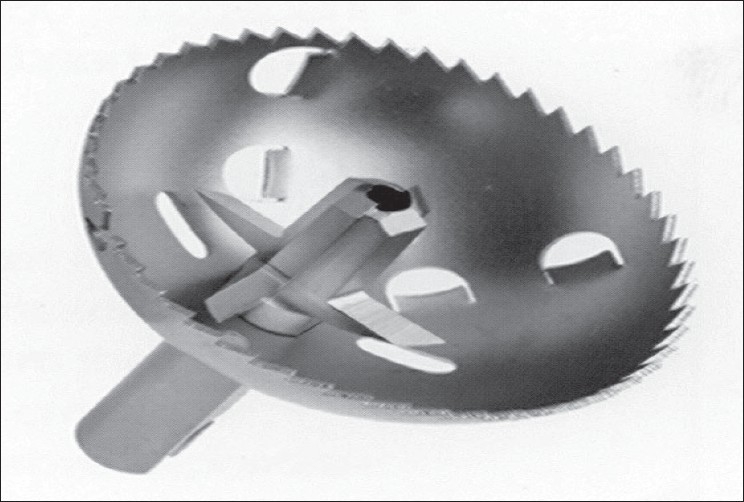
Global CAP sharper

We can attribute this complication to the osteopenic bone of the patient, but also to an excessive reaming (possibly and excessive strength was applied) or to the characteristics of the humeral head shaper design, that could be more aggressive that the Copeland's one [Figure 3]. Therefore, in order to avoid our perioperative complication, we suggest that a very cautious reaming of the humeral head must be done when a Resurfacing humeral head is implanted. Nevertheless, this prosthesis could be a useful treatment, also in elderly people. However, it is advisable that conventional stemmed implants should be available in the case that a RRH is performed.
